# The Skin-Whitening Effects of Ectoine via the Suppression of α-MSH-Stimulated Melanogenesis and the Activation of Antioxidant Nrf2 Pathways in UVA-Irradiated Keratinocytes

**DOI:** 10.3390/antiox9010063

**Published:** 2020-01-10

**Authors:** You-Cheng Hseu, Xuan-Zao Chen, Yugandhar Vudhya Gowrisankar, Hung-Rong Yen, Jing-Yuan Chuang, Hsin-Ling Yang

**Affiliations:** 1 Department of Cosmeceutics, College of Biopharmaceutical and Food Sciences, China Medical University, Taichung 40402, Taiwan; ychseu@mail.cmu.edu.tw (Y.-C.H.);; 2 Department of Health and Nutrition Biotechnology, Asia University, Taichung 41354, Taiwan; 3 Chinese Medicine Research Center, China Medical University, Taichung 40402, Taiwan; 4 Research Center of Chinese Herbal Medicine, China Medical University, Taichung 40402, Taiwan; 5 Department of Medical Research, China Medical University Hospital, Taichung 40402, Taiwan; 6 School of Chinese Medicine, China Medical University, Taichung 40402, Taiwan; 7 Department of Medical Laboratory Science and Biotechnology, China Medical University, Taichung 40402, Taiwan; 8 Institute of Nutrition, College of Biopharmaceutical and Food Sciences, China Medical University, 91 Hsueh-Shih Road, Taichung 40402, Taiwan

**Keywords:** Ectoine, keratinocytes, melanogenesis, tyrosinase, α-MSH, Nrf2

## Abstract

Ultraviolet A (UVA)-irradiation induced reactive oxygen species (ROS) production mediates excessive melanogenesis in skin cells leading to pigmentation. We demonstrated the depigmenting and anti-melanogenic effects of Ectoine, a natural bacterial osmolyte, in UVA-irradiated human (HaCaT) keratinocytes, and the underlying molecular mechanisms were elucidated. HaCaT cells were pre-treated with low concentrations of Ectoine (0.5–1.5 μM) and assayed for various depigmenting and anti-melanogenic parameters. This pre-treatment significantly downregulated ROS generation, α-melanocyte-stimulating hormone (α-MSH) production, and proopiomelanocortin (POMC) expression in UVA-irradiated HaCaT cells. Also, antioxidant heme oxygenase-1 (HO-1), NAD(P)H dehydrogenase [quinone 1] (NQO-1), and γ-glutamate-cysteine ligase catalytic subunit (γ-GCLC) protein expressions were mediated via the nuclear translocation of nuclear factor erythroid 2-related factor 2 (Nrf2) whose knockdown indeed impaired this effect signifying the importance of the Nrf2 pathway. Ectoine was mediating the activation of Nrf2 via the p38, protein kinase B (also known as AKT), protein kinase C (PKC), and casein kinase II protein kinase (CKII) pathways. The conditioned medium obtained from the Ectoine pre-treated and UVA-irradiated HaCaT cells downregulated the tyrosinase, tyrosinase-related protein-1 and -2 (TRP-1/-2), cyclic AMP (c-AMP) protein kinase, c-AMP response element-binding protein (CREB), and microphthalmia-associated transcription factor (MITF) expressions leading to melanoma B16F10 cells having inhibited melanin synthesis. Interestingly, this anti-melanogenic effect in α-MSH-stimulated B16F10 cells was observable only at 50–400 μM concentrations of Ectoine, signifying the key role played by Ectoine (0.5–1 μM)-treated keratinocytes in skin whitening effects. We concluded that Ectoine could be used as an effective topical natural cosmetic agent with depigmenting and anti-melanogenic efficacy.

## 1. Introduction

Exposing human skin to UVA radiation triggers ROS generation and also over-producing melanin in the skin cells. The uncontrolled production of ROS could lead to melanoma conditions as well. Most skin whitening agents are targeting and trying to minimize the melanogenesis process through the inhibition of α-MSH and tyrosinase productions [1]. Most skin-tone lightening creams are composed of hydroquinone [2] or hydrocortisone [3], that are known to decrease the formation of melanin, but are also associated with severe side effects. For example, acne, flaky and itchy skin, blue and black discoloration of the skin, ochronosis, burning and stinging, skin irritation, and even inflammation. However, skin whitening agents from the natural sources, for example, Kojic acid (a fungal derivative obtained from *Penicillium* and *Aspergillus* species) is also reported to cause ‘contact dermatitis’ in individuals who have sensitive skin. In these individuals, more than 1% of kojic acid could cause severe hypersensitive side effects [4,5]. Therefore, only a few naturally derived skin whitening products (oleosin, licorice extract, ascorbic acid, soy protein, and *N*-acetyl glucosamine, etc.) are currently being used in the cosmetic industry [6]. However, the skincare products that are principally targeting the depigmenting properties have at times failed to focus on counteracting the deleterious effects posed by the UVA irradiation-induced ROS production mediated excess melanogenesis in skin cells.

Ectoine is a ‘natural extremolyte’ produced from several species of microorganisms under stressful conditions [7,8]. This compound was first isolated from the *Ectothiorhodospira* species of bacteria that are living in the Egyptian desert. The cascade of *ect* operon genes (*ect*A, *ect*B, *ect*C, or *ect*D) are involved in the production of this compound. Ectoine is chemically designated as 1,4,5,6-tetrahydro-2-methyl-4-pyrimidinecarboxylic acid [9]. As a moisture binder, Ectoine helps in, restructuring of the skin cell membrane [10], protection from UV damage and pollution [11,12], moisturizing the skin [13], delaying the premature skin aging [14], etc. In addition to the skin protective roles, Ectoine has been shown to be useful in the treatment of atopic dermatitis [15], Alzheimer’s [16], as well as the inhibition of HIV replication [17], radio and chemotherapy [18], and liver cirrhosis [19]. Ectoine is speculated to exhibit its depigmenting and skin whitening properties without causing undesirable side effects [20]. Contrastingly, the molecular mechanisms elicited by Ectoine are not known. Therefore, the objective of this study was to delineate the Ectoine mediated depigmenting and anti-melanogenic mechanisms elicited in UVA-irradiated human (HaCaT) keratinocytes as the cellular model system. The effect of Ectoine induced secretions of skin-protecting agents from the HaCaT cells to the culture medium (conditioned medium) was also tested using a typical melanoma cell (B16F10) line as well.

## 2. Materials and Methods

### 2.1. Reagents and Antibodies

Ectoine (Product no: 81619, purity ≥ 95%) was purchased from Sigma-Aldrich (Taufkichen, Germany). Fetal bovine serum (FBS), penicillin/streptomycin, Dulbecco’s modified Eagle’s medium (DMEM) and l-glutamine were bought from Invitrogen/Gibco BRL (Carlsbad, CA, USA). l-DOPA, melanin, 3-4,5-dimethyl-2-yl-2,5-diphenyl tetrazolium bromide (MTT), and α-MSH were procured from Sigma Chemical Co (St. Louis, MO, USA). *N*-acetylcysteine (NAC) and 2′,7′-dichlorofluorescein-diacetate (DCFH_2_-DA) were procured from Sigma-Aldrich (St. Louis, MO, USA). All pharmacological inhibitors required for JNK (SP600125), ERK1/2 (PD98059), p38 (SB203580), PKC (GF109203X), and CKII were obtained from Calbiochem (La Jolla, CA, USA) PI3K/AKT inhibitor (LY294002) was obtained from Sigma-Aldrich (St. Louis, MO, USA). All antibodies for POMC, CREB, β-actin, tyrosinase, Nrf2, p-CREB, NQO-1, PKC, Kelch-like ECH-associated protein-1 (Keap-1), and TRP-1, TRP-2 were obtained from Santa Cruz Biotechnology Inc. (Heidelberg, Germany). Antibodies against γ-GCLC and HO-1 were procured from Gene Tex Inc. (San Antonio, TX, USA). We obtained antibodies against c-AMP protein kinase and CKII from Abcam (Cambridge, MA, USA). Histones, MITF, p-p38, p38, p-AKT, and AKT were obtained from Cell Signal Technology (Beverly, MA, USA). Enhanced chemiluminescence (ECL) detection reagents were obtained from Millipore, (Billerica, MA, USA). All other reagents (at HPLC grade) were either purchased from Sigma-Aldrich or Merck & Co., Inc. (Darmstadt, Germany).

### 2.2. Cell Culture

We obtained immortalized human skin keratinocyte HaCaT and murine melanoma B16F10 cells from both Cell Line Services (CLS, Eppelheim, Germany) and American Type Culture Collection (ATCC, VA, USA). The cells were cultured in DMEM supplemented with 10% heat-activated FBS, 1% streptomycin/penicillin, and 2 mM L-glutamine in a humidified incubator supplemented with 5% CO_2_ at 37 °C.

### 2.3. Cell Treatments and UVA-Irradiation

Before UVA irradiation, the cells were pre-treated with Ectoine (0.5–1.5 μM for 24 h) or vehicle (PBS). Post incubation, PBS washed cells were exposed to 3 J/cm^2^ UVA radiations (for 27 min, λ_max_, 365 nm, no detectable emissions below 320 nm) using the UV CROSS-LINKER CL-508 (UVItec, Cambridge, UK) [21].

### 2.4. Cell Viability Assay

HaCaT cells (5 × 10^4^ cells/well) were seeded in a 24-well plate containing DMEM and supplemented with 10% FBS and varying concentrations of Ectoine (0.5–1.5 μM, 24 h). Then it was irradiated in the absence or presence of UVA (3 J/cm^2^). B16F10 cells (5 × 10^4^ cells/well) were seeded in a 24-well plate containing DMEM and supplemented with 10% FBS and varying concentrations of Ectoine (100–400 μM, 72 h). The cells were washed with PBS and an MTT cell viability assay was conducted [21].

### 2.5. Intracellular ROS Assay

HaCaT cells were seeded at a density of 1.5 × 10^5^ in 8-well Lab Teck chamber containing DMEM supplemented with 10% FBS and were grown to 80% confluence. These cells were first treated with 1.5 μM Ectoine, followed by exposure to 3 J/cm^2^ UVA irradiation for the prescribed amount of time. Cells were washed with PBS and DCFH_2_-DA method was used to determine the intracellular ROS production using the Olympus Software solution software for each condition [21].

### 2.6. Melanin Quantification

In a 6-well plate, murine melanoma B16F10 cells were seeded at a density of 2.5 × 10^5^ cells/well. They were pre-treated with 100, 200, and 400 μM of Ectoine for 2 h in the absence or presence of α-MSH (1 μM). The protocol used for the quantification of melanin followed a previously described method [21]. We measured melanin content with the ELISA microplate reader with an absorbance wavelength of 470 nm.

### 2.7. Western Blot

HaCaT (1 × 10^6^ cells/10 cm dish) or B16F10 (1 × 10^6^ cells/10 cm dish) cells were pre-treated with varying concentrations of Ectoine (0.5, 1, and 1.5 μM) or α-MSH (1 μM) followed by irradiation in the absence or presence of UVA for the prescribed amount of time. PBS washed cells were harvested, the protein content (nuclear and cytosolic) was isolated after treatment. Then, the cells were subjected to the Western blot method used previously for the determination of expressions of various nuclear and cytosolic proteins [21].

### 2.8. RNA Extraction and RT-PCR

Ectoine pre-treated (1.5 μM, 24 h) HaCaT cells were subjected to the TRIzol reagent (Invitrogen, Carlsbad) for the isolation of total RNA from these cells. 1 µg of total RNA and the reagents supplied by the SuperScript-III One-Step RT-PCR platinum *Taq* kit (Invitrogen, Carlsbad) were used in the PCR experiment with the Bio-Rad iCycler PCR instrument (Bio-Rad, Hercules, CA, United States). The forward and reverse primers for Nrf2 used were: F: 5′-AAACCAGTGGATCTGCCAAC-3′, R-5′-GCAATGAAGACTGGGCTCTC-3′. The forward and reverse primers for GAPDH used were: F: 5′-GCATCCTGGGCTACACTGA-3′, R: 5′-CCACCACCCTGTTGCTGTA-3′. At the end of the experiment, PCR product was analyzed using 1% agarose gel. Then, it was visualized with ethidium bromide staining. As an internal control, we used GAPDH [22].

### 2.9. Immunofluorescence Assay

HaCaT cells were cultured at a density of 1 × 10^4^ cells/well in DMEM supplement with 10% FBS in an 8-well Lab Tek chamber (Thermo Fisher Scientific, Waltham, MA, USA). We pretreated the cells with 1.5 μM Ectoine for the indicated time followed by irradiation in the absence or presence of UVA. The cells were subjected to an immunofluorescence assay, which uses a method previously described [21].

### 2.10. siRNA Transfection

For siRNA transfection, HaCaT cells were plated in a 6-well plate and were grown till it has reached a confluence of 40–60% at the time of transfection. The remaining protocol was followed according to a method that was explained before [21].

### 2.11. Statistical Analysis

We used the mean ± standard deviation (mean ± SD) for all the results used in this study. All data were analyzed with an analysis of variance (ANOVA), followed by Dunnett’s test for pair-wise comparisons and presented as mean ± SD of three or more independent experiments. Statistical significance was set at * *p* < 0.05; ** *p* < 0.01; *** *p* < 0.001 when compared with untreated control cells, and ^#^ *p* < 0.05; ^##^ *p* < 0.01; ^###^ *p* < 0.001 when compared with the UVA-exposed HaCaT cells or α-MSH treated B16F10 cells.

## 3. Results

### 3.1. Ectoine Inhibited UVA-Induced ROS Generation in HaCaT Cells

**Figure 1:**
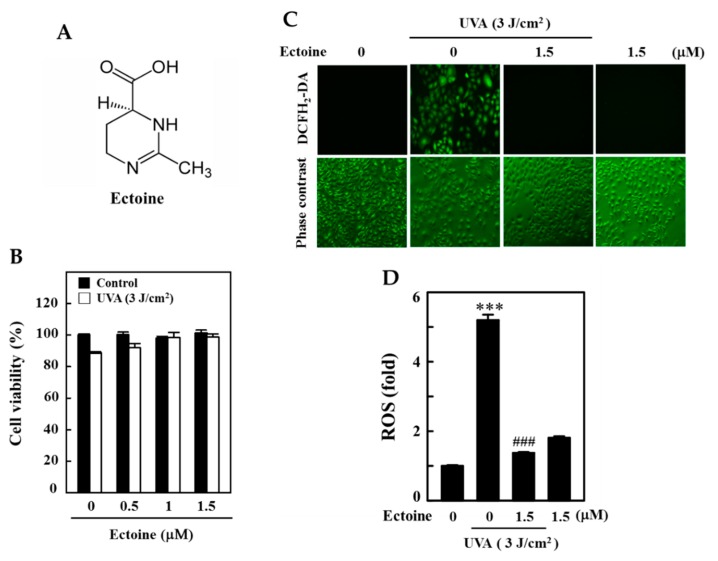
Ectoine inhibits UVA-induced ROS production in human keratinocyte (HaCaT) cells. (**A**) Ectoine’s chemical structure. (**B**) To determine cell viability, an MTT assay was used. Cells were treated with Ectoine (0.5, 1, and 1.5 μM) for 24 h. Then, they were irradiated with 3 J/cm^2^ or without UVA. (**C**,**D**) Cells were pre-treated with Ectoine (0 or 1.5 μM) for 24 h and then irradiated with 3 J/cm^2^ or without UVA. For each condition, we used the percentage of the fluorescence intensity of the DCF-stained cells as determined by Olympus Softimage. Statistical significance was assigned as *** *p* < 0.001 compared to the untreated control cells and ^###^ *p* < 0.001 compared to the UVA-exposed HaCaT cells.

First, we tested for the cytotoxic effects of Ectoine (Figure 1A) on UVA-irradiated HaCaT cells. Our MTT data indicated that when compared to the untreated control cells, Ectoine pre-treated (0.5–1.5 μM) and 3 J/cm^2^ UVA exposed HaCaT cells were unable to show a significant decrease in cell viability (Figure 1B). Further, Ectoine pretreatment attenuated the UVA (3 J/cm^2^)-induced cell death in a dose-dependent manner (Figure 1B). In addition to our fluorescence data, which indicated that, when compared to the control cells, 3 J/cm^2^ UVA irradiation and Ectoine alone treatments (1.5 μM) significantly upregulated ROS levels by 5- and 2-fold, respectively. However, in the case of Ectoine pretreatment ROS levels were significantly downregulated and we can infer that Ectoine has an antioxidant effect against UVA irradiation. This also induces basal levels of ROS in HaCaT cells (Figure 1C,D).

### 3.2. Ectoine Suppressed POMC and α-MSH Expressions in UVA-Irradiated HaCaT Cells

**Figure 2:**
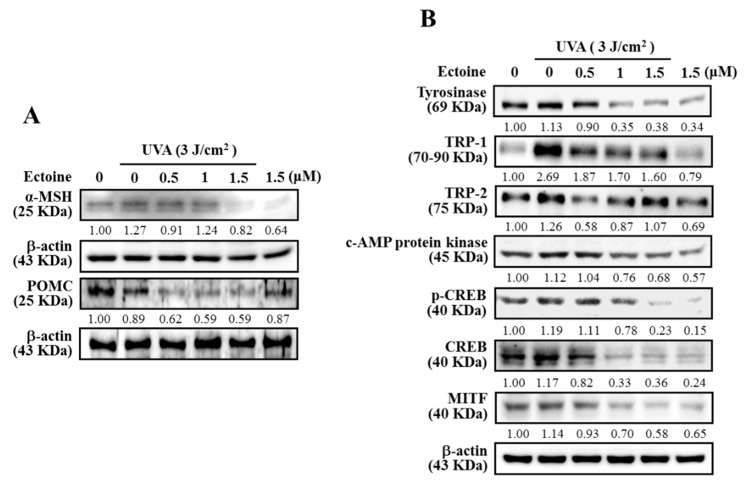
Ectoine suppresses UVA-induced POMC and α-MSH expression in HaCaT cells. (**A**) Western blotting showed us protein levels of α-MSH and POMC. Cells were pre-treated with Ectoine (0–1.5 μM) for 24 h and then irradiated or not with 3 J/cm^2^ UVA. (**B**) Effect of HaCaT conditioned medium on B16F10 cells melanin synthesis. HaCaT cells were pre-treated with vehicle (PBS) or Ectoine (0.5–1.5 μM) for 24 h. Subsequently, these cells were exposed or non-exposed to the 3 J/cm^2^ UVA-irradiation. After 1–24 h, the conditioned medium (10 mL/100 mm dish) was collected and tested on B16F10 cells for the expression of various proteins through western blot method. Lanes 1 and 2 were indicating the experimental conditions of conditioned medium obtained from vehicle (PBS) pre-treated and UVA non exposed (lane #1) and exposed (lane #2). Whereas, lanes 3–5, and 6 were indicating the experimental conditions of conditioned medium obtained from Ectoine pre-treated (0.5–1.5 μM) and UVA exposed, and non-exposed, respectively. Western blot analysis measured the protein levels of tyrosinase, TRP-1, TRP-2 (for 24 h), c-AMP protein kinase (for 1 h), p-CREB, CREB (for 2 h), and MITF (for 4 h).

UVA exposed keratinocytes were stimulated for their ROS-p53 mediated POMC and also a small peptide hormone α-MSH that is derived from POMC [23]. Therefore, we determined the alterations in expression patterns of α-MSH, POMC, and other associated proteins in Ectoine pre-treated HaCaT cells and then exposed them to UVA (3 J/cm^2^). Western blot data indicated that UVA-induced upregulation of α-MSH and POMC expressions were downregulated by Ectoine pretreatment; whereas, Ectoine treatment without UVA irradiation has completely inhibited the α-MSH and POMC expressions of non-irradiated HaCaT cells (Figure 2A). Later, we tested the effect of ‘conditioned-medium’ (10 mL/100 mm plate), obtained from the Ectoine pre-treated and UVA irradiated HaCaT cells, on the melanogenesis of B16F10 melanoma cells. Figure 2B shows this conditioned medium downregulated the tyrosinase, TRP-1, TRP-2, c-AMP protein kinase, p-CREB, CREB, and MITF levels in B16F10 cells.

### 3.3. Ectoine Downregulated Melanin and Tyrosinase Expression in α-MSH-Stimulated B16F10 Cells

**Figure 3:**
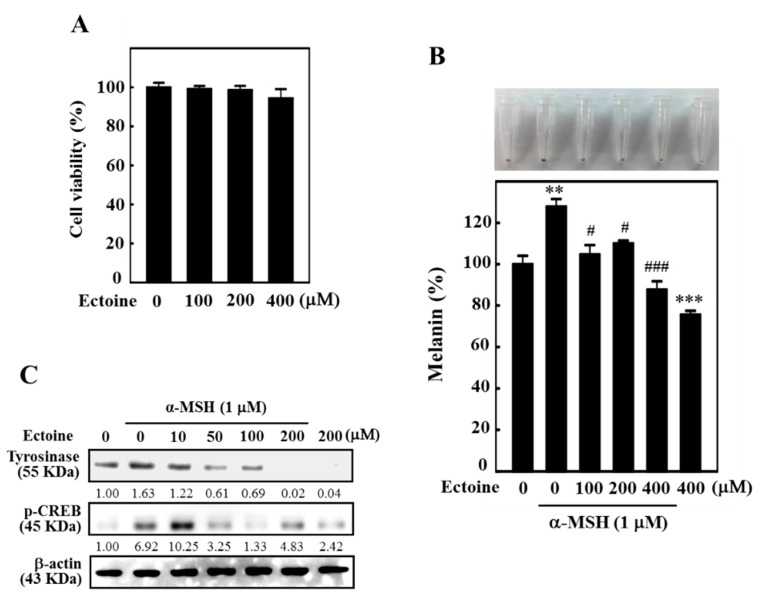
Ectoine downregulated the melanogenesis in α-MSH-stimulated B16F10 cells. (**A**) High concentrations of Ectoine (100–400 μM, 72 h) affect the cell viability of B16F10 as determined by an MTT assay. (**B**) Cells were pre-treated with Ectoine (0–400 μM, 1 h) followed by stimulation without or with 1 μM α-MSH for 72 h. The percentage of melanin content was quantified from total cell lysates. (**C**) Cells were pre-treated with Ectoine (0–200 μM for 1 h) and then treated with or without α-MSH (1 μM) for 24 or 2 h. Western blot analysis measured the expressions of tyrosinase and p-CREB proteins. Statistical significance was assigned as ** *p* < 0.01; *** *p* < 0.001 compared to the untreated control cells and ^#^ *p* < 0.05, ^###^ *p* < 0.001 compared to the α-MSH treated B16F10 cells.

B16F10 melanoma cells were first subjected to the higher concentrations of Ectoine and the effect of cytotoxicity was determined using MTT assay. Figure 3A shows that Ectoine had no significant impact on the viability of B16F10 cells at higher concentrations (100–400 µM for 72 h). However, cell viability was not effected at 24 and 48 h of Ectoine treatment (data not shown). Therefore, these concentrations were used to determine the effect of Ectoine on α-MSH-stimulated melanogenesis in B16F10 cells. Melanin quantification data showed that, compared to the control cells, treatment with α-MSH (1 µM) alone significantly upregulated the melanin levels by more than 25%. However, compared to α-MSH alone treatment, cells exposed to increasing concentrations of Ectoine (100–400 µM at 72 h) dose-dependently and significantly downregulated the percentage of melanin content with maximum downregulation of only 85% (or −15% than untreated control) was observed at 400 µM of Ectoine pretreatment (Figure 3B). Moreover, our Western blot data also showed that α-MSH stimulated tyrosinase (24 h) and p-CREB (2 h) expressions were significantly downregulated with increasing concentrations of Ectoine pretreatments in these melanoma cells (Figure 3C).

### 3.4. Ectoine Facilitated Nrf2 Nuclear Translocation in HaCaT Cells

**Figure 4:**
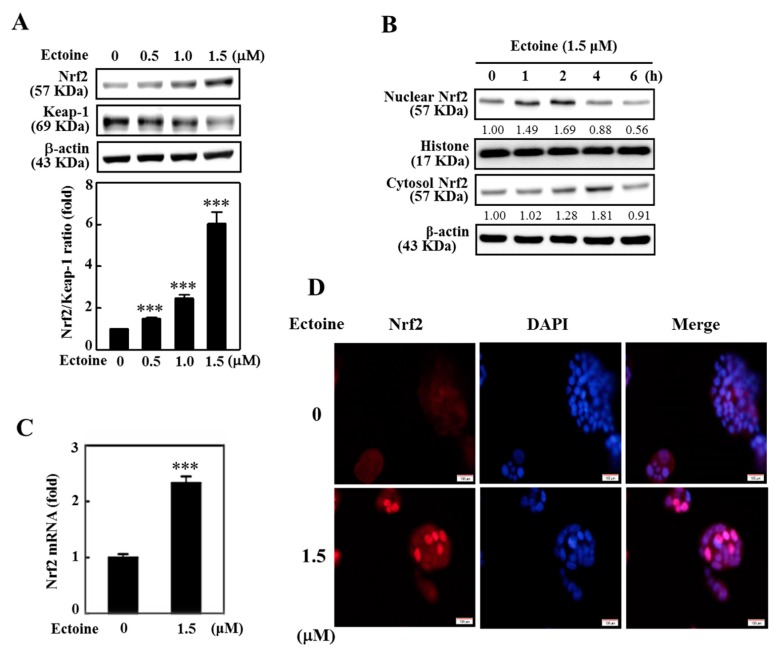
Ectoine upregulated the nuclear translocation of antioxidant marker Nrf2 in HaCaT cells. (**A**) Effect of Ectoine on protein expressions of Nrf2 and Keap-1 in HaCaT cells. Western blot method measured the expressions of Nrf2 and Keap-1 in cells treated with Ectoine (0–1.5 μM) for 2 h (**B**) Effect of time on the Ectoine mediated nuclear and cytosolic expressions of Nrf2. Cells were exposed to 1.5 μM Ectoine for different time points and Western blot method measured the expressions of nuclear and cytosolic Nrf2. (**C**) Cells were pre-treated with Ectoine (1.5 μM, 1 h) followed by the isolation of total mRNA from HaCaT cells. 1 μg of total mRNA was used to measure the expression of the Nrf2 gene through the RT-PCR method. As an internal control, GAPDH was used. (**D**) Immunofluorescence staining of HaCaT cells. Cells were treated with 1.5 μM of Ectoine for 2 h and the nuclear localization of Nrf2 were visualized by immunofluorescence method. Cells were stained with DAPI (1 μg/mL) for 5 min and examined by fluorescence microscopy (scale bar 100 µm). Statistical significance was assigned as *** *p* < 0.001 compared to the untreated control cells.

Nrf2-Keap-1 is an important cytoprotective pathway that protects the skin cells from ROS and electrophile insult caused by UVA exposure. Nrf2 and Keap1 maintain a stoichiometric ratio in the cytoplasm. In a cytoprotective pathway, Nrf2 dissociates from Keap-1 and, for the expression of various antioxidant genes, translocate to the cellular nucleus. Therefore, the ratio of Nrf2/Keap-1 is a key factor. Here, our Western blot data indicated a shift in the ratio of Nrf2/Keap-1 that was towards more Nrf2 expression with the increasing concentrations of Ectoine, signifying that Ectoine favors the nuclear translocation of Nrf2 in HaCaT cells (Figure 4A,B). Also, the expression of Nrf2 mRNA levels was shown to be significantly elevated in the 1.5 μM Ectoine pre-treated cells (Figure 4C). This was also consistent with our fluorescence image data (Figure 4D).

### 3.5. Ectoine Upregulated the Expression of HO-1, NQO-1, and γ-GCLC Proteins in HaCaT Cells

**Figure 5:**
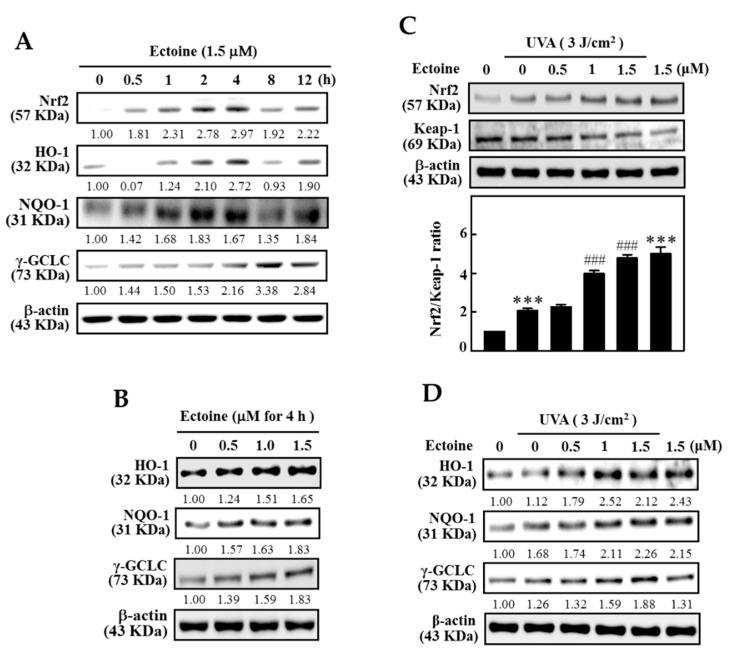
Ectoine mediated differential expressions of antioxidant genes in UVA irradiated HaCaT cells. (**A**,**B**) Western blot method was used to determine the effects of time (0–12 h) and Ectoine concentrations (0–1.5 μM) on the expressions of Nrf2 and antioxidant genes (HO-1, NQO-1, and γ-GCLC) in HaCaT cells. (**C**,**D**) Effect of Ectoine pretreatment effect on the Nrf2/Keap-1 ratio of HaCaT cells irradiated with UVA. Cells were pre-treated with Ectoine (0–1.5 μM for 24 h), and then irradiated in the absence or presence of 3 J/cm^2^ UVA. The ratio of Nrf2/Keap-1 (**C**) and antioxidant gene (HO-1, NQO-1, and γ-GCLC) expressions (**D**) were determined by Western blotting. Statistical significance was assigned as *** *p* < 0.001 compared to the untreated cells and ^###^ *p* < 0.001 compared to the UVA exposed HaCaT cells.

To determine the effect of time on Ectoine mediated nuclear translocation of Nrf2 and the subsequent downstream expression of HO-1, NQO-1, and γ-GCLC proteins, HaCaT cells were exposed to 1.5 µM Ectoine and the cellular proteins were harvested 0.5, 1, 2, 4, 8, or 12 h after the Ectoine treatment. Western blot data indicated that, except for the γ-GCLC protein, 1.5 μM Ectoine caused the maximum expression of HO-1, Nrf2, and NQO-1 proteins at the 4 h time point. γ-GCLC was shown at 8 h time point (Figure 5A). Data obtained from the time-curve lead us to test the effect of Ectoine concentration on the expression of antioxidant proteins at 4 h time point. Figure 5B shows that all three antioxidant proteins exhibited maximum expression at 1.5 µM of Ectoine concentration. Later, the effects of Ectoine pre-treatment were tested on the expression of Nrf2 and Keap-1 ratio in the UVA-irradiated HaCaT cells. Western data analysis indicated that pre-treatment with 1.5 µM of Ectoine exhibited an increase in the ratio of Nrf2/Keap-1 in UVA-irradiated HaCaT cells (Figure 5C). We also saw consistent data with the increased expression of NQO-1, HO-1, and γ-GCLC proteins in Ectoine pre-treated HaCaT cells that were irradiated with 3 J/cm^2^ UVA (Figure 5D). This data infers that Ectoine pre-treatment plays a protective role in UVA-irradiated HaCaT cells.

### 3.6. Various Signaling Pathways Were Involved in the Activation of Nrf2 in Ectoine Treated HaCaT Cells

**Figure 6:**
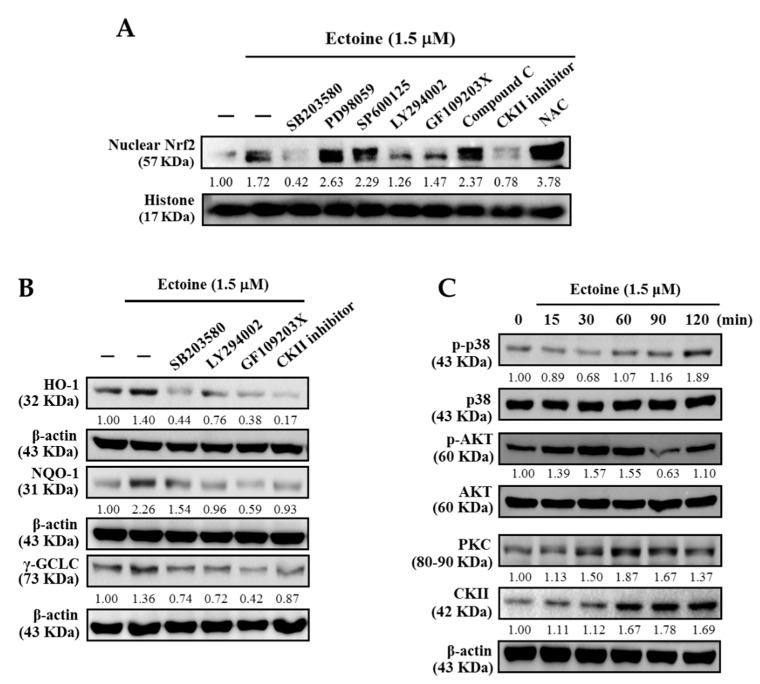
Ectoine mediated the activation of nuclear Nrf2 through p38, AKT, PKC, and CKII signaling pathways in HaCaT cells. (**A**) Cells were pre-treated with p38 inhibitor (SB203580, 20 μM), ERK inhibitor (PD98059, 30 μM), JNK inhibitor (SP600125, 25 μM), PI3K/AKT inhibitor (LY294002, 30 μM), PKC inhibitor (GF109203X, 2.5 μM), AMPK inhibitor (compound C, 10 μM), Casein kinase II inhibitor (CKII, 20 μM), or antioxidant NAC (1 mM) for 30 min, followed by exposure to Ectoine (1.5 μM) for 2 h. Western blot was performed to analyze the nuclear Nrf2 expression against histone proteins as internal control (**B**) HO-1, NQO-1, and γ-GCLC protein levels were evaluated using immunoblot analysis. Cells were pre-treated with inhibitors for p38 (SB203580, 20 μM), PI3K/AKT (LY294002, 30 μM), PKC (GF109203X, 2.5 μM), and CKII (20 μM) for 30 min followed by exposure to Ectoine (1.5 μM) for 4–8 h. (**C**) Ectoine activated the p38, AKT, PKC, and CKII signaling pathways. Cells were pre-treated with Ectoine (1.5 μM) for 0–120 min, and the protein expressions of p-p38, p38, p-AKT, AKT, PKC, and CKII were measured by immunoblot analysis.

We determined the signaling pathways involved in the Ectoine mediated nuclear translocation of Nrf2. HaCaT cells were pre-treated with pharmacological inhibitors of PI3K/AKT, ERK, p38, JNK, PKC, ROS, and CKII signaling pathways, followed by 1.5 μM Ectoine. Western blot data of nuclear Nrf2 showed that p38 MAPK, PI3K/AKT, PKC, and CKII pathways were involved in this mechanism (Figure 6A). From the obtained information, we also determined the effect of Ectoine pre-treatment on the role played by these pathways in the expression of antioxidant proteins. Figure 6B shows that pharmacological inhibition of MAPK, p38, PI3K/AKT, CKII, and PKC pathways down-regulated the expression of NQO-1, HO-1, and γ-GCLC antioxidant proteins in HaCaT cells. Moreover, the time taken for the phosphorylation of AKT, p38, and the expression of PKC and CKII while exposed to Ectoine indicates that, except for the p-AKT, the phosphorylation of p38 and the expressions of PKC and CKII took place at the later time points only (after 30 min) (Figure 6C). In the case of AKT, phosphorylation was observed from the 15 min time point that has reached a peak at 30 min (Figure 6C). These cumulative results suggested that p38, AKT, PKC, and CKII signaling pathways activated the Ectoine mediated nuclear translocation of Nrf2 leading to the expression of antioxidant proteins.

### 3.7. Ectoine Mediated Anti-Melanogenic Effect was Suppressed due to the Knockdown of Nrf2

**Figure 7:**
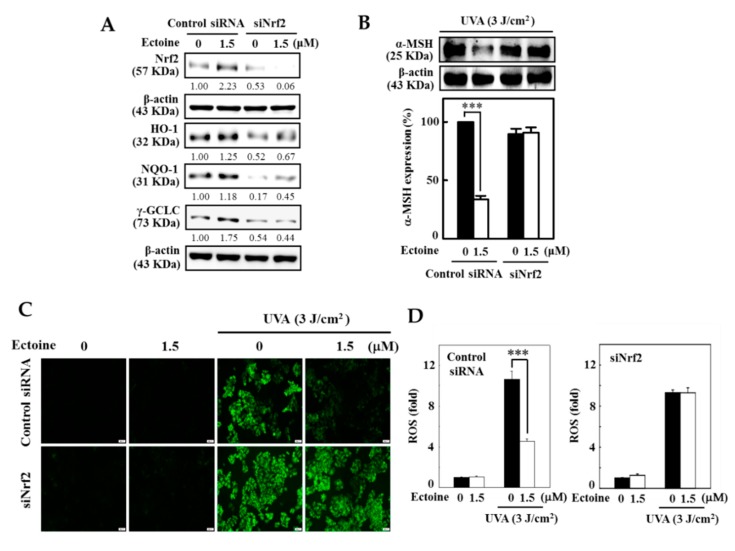
Nrf2 knockdown attenuated the protective effects of Ectoine in UVA-irradiated HaCaT cells. Cells were transfected with siRNA that is specific to either Nrf2 or a non-silencing control. (**A**) Transfected cells were pre-treated with Ectoine (0 or 1.5 μM for 2 or 12 h) and the expression of Nrf2 (for 2 h), or HO-1, NQO-1, and γ-GCLC (for 12 h) proteins in both control and siNrf2 were measured by Western blot analysis. (**B**) The effect of Nrf2 knockdown on the expression of α-MSH levels in UVA irradiated HaCaT cells were determined. Transfected cells were pre-treated with or without Ectoine (1.5 μM for 24 h) and then irradiated with 3 J/cm^2^ UVA. The Western blot method measured the percentage of α-MSH levels (**C**,**D**) The effect of Nrf2 knockdown on the UVA radiation-induced ROS levels in transfected cells were measured by DCF fluorescence microscopy. Statistical significance was assigned as *** *p* < 0.001 compared to the untreated cells.

The role of Nrf2 in Ectoine mediated anti-melanogenesis was determined by silencing the Nrf2 in HaCaT cells. Data from the Western blot indicated that Nrf2 knockdown cells exposed to 1.5 μM Ectoine showed minimum expression of NQO-1, HO-1, and γ-GCLC antioxidant proteins (Figure 7A). Later, we tested the effect of Nrf2 knockdown on the expression of α-MSH levels in UVA irradiated (3 J/cm^2^) HaCaT cells. Western blot results indicated that to control the siRNA transfected cells, UVA-irradiation was significant in the upregulation of the expression of α-MSH levels in cells unexposed to Ectoine (Figure 7B). However, 1.5 μM Ectoine has suppressed this effect. For the other case, cells transfected with siNrf2 showed a decrease in the expression of α-MSH levels in both untreated and treated cells (Figure 7B). Similar to α-MSH data, our fluorescence data also indicated that UVA irradiation significantly upregulated the ROS production in Ectoine untreated control siRNA cells (Figure 7C,D). However, this effect was significantly suppressed when the cells exposed to 1.5 μM Ectoine. On the other hand, Nrf2 transfected and UVA-irradiated HaCaT cells showed an approximately 8-fold increase in ROS levels compared to the Nrf2 transfected cells that were not irradiated with UVA but exposed to Ectoine treatment (Figure 7C,D). All this data signifies the Ectoine mediated protective role played by Nrf2 in the minimization of melanin production in UVA-irradiated HaCaT cells.

## 4. Discussion

Various skin-whitening agents are in use in the cosmetic industry. Many of these agents are from the chemical origin and are suffering from the limitations of causing various side effects including the cancers [24,25,26]. Therefore, the identification of safe and natural skin-whitening agents represent the need of the hour. Ectoine (Figure 1A) has been known to be used as an active ingredient in face creams and other cosmetic agents. This acts as a skin moisturizing agent and also considered to delay the premature skin-aging as well [27]. Almost all known skin-whitening agents target the downregulation of tyrosinase enzyme activity in UV-irradiated cells that decreases the melanogenesis in skin cells. Yao et al. demonstrated the whitening properties of biosynthesized Ectoine and suggested that it is a putative whitening agent. In their study, they tested the high concentration (500 µM) of Ectoine for its whitening effect on mouse melanoma (B16F0) and human melanoma (A2058) cell lines and concluded that Ectoine is a safe and potential agent for the cosmetic and clinical application [20]. However, in this study, we further tested the beneficial effects of low concentrations of Ectoine (0.5–1.5 µM) on UVA-irradiated HaCaT cells and the underlying molecular mechanisms were deciphered. In our study, it was shown that Ectoine, through the Nrf2/ARE pathway, has not only induced the expression of antioxidant gene expression but also downregulated the α-MSH levels in UVA-irradiated HaCaT cells via the suppression of POMC. A decrease in the α-MSH levels was correlated with downregulation of tyrosinase enzyme activity leading to the decrease in the melanin production. From our knowledge, this is first the report that was evidenced by the mechanism elicited by Ectoine in UVA-irradiated HaCaT cells. This study delineated the molecular mechanisms exhibited by Ectoine in HaCaT cells as the cellular model system.

We first determined the sub-lethal concentrations of Ectoine as well as the effect of UVA radiation on the viability of HaCaT cells. Our MTT data indicated that low concentrations of Ectoine (0.5–1.5 µM) had no significant effect on the viability of HaCaT cells (Figure 1B). Ectoine pre-treatment increased the viability of 3 J/cm^2^ UVA-irradiated HaCaT cells (Figure 1B). Based on these observations, we continued our further experiments using 1.5 µM of Ectoine pre-treatment and UVA irradiation at 3 J/cm^2^ dosage.

UVA irradiation-induced ROS production in skin keratinocytes is a well-known fact [28]. Therefore, we also tested for any beneficial effects from Ectoine pre-treatment in the UVA-radiation induced ROS production in HaCaT cells. Our DCF-fluorescence intensity data indicated that pre-treatment with 1.5 µM of Ectoine significantly downregulated the UVA-radiation induced ROS production in keratinocytes. It was also observable that 1.5 µM of Ectoine could cause a basal level increase in the ROS levels in HaCaT cells that were shown to be statistically significant (Figure 1D,E).

Rousseau et al. reported that POMC, is secreted by human epidermal keratinocytes and melanocytes and has stimulated the melanogenesis [29]. By keeping this in view, we too tested the effect of UVA irradiation and Ectoine pre-treatments on the melanogenesis associated proteins in HaCaT cells. Our Western blot data indicated the dose-dependent downregulation of the expression of α-MSH and POMC proteins in UVA-irradiated HaCaT cells was caused by the pretreatment by Ectoine. Conversely, Ectoine pre-treatment has a differential effect on the expression pattern of melanogenesis associated proteins. Notably, almost all tested proteins (Tyrosinase, TRP-1, TRP-2, c-AMP protein kinase, CREB, and MITF) showed decreased expressions with increasing concentrations of Ectoine pre-treatment in UVA-irradiated HaCaT cells (Figure 2A,B). This data signifies the fact that Ectoine possesses anti-melanogenic properties in UVA-irradiated HaCaT cells.

The anti-melanogenic efficacy of Ectoine was further tested in B16F10 cells, a well-known melanoma cell line used in melanogenesis studies [30]. One of the notable observations in our study was that, in contrast to the HaCaT cells, high concentrations of Ectoine (100–400 μM) were necessary to suppress the melanin synthesis in α-MSH-stimulated B16F10 cells (Figure 3B). Our Western blot data indicated that Ectoine dose-dependently downregulated the expression of tyrosinase and p-CREB proteins in α-MSH-stimulated B16F10 cells, leading to the aforesaid effect (Figure 3C). Therefore, we also tested if these high concentrations of Ectoine could affect the viability of B16F10 cells. Our MTT results indicated that high concentrations of Ectoine (100–400 μM) had no effect on the viability of B16F10 cells (Figure 3A). These results signify that keratinocytes play a key role in Ectoine mediated anti-melanogenesis and depigmenting effects.

The role of transcription factor Nrf2 in skin cells metabolism was well documented [31]. Therefore, we further tested the mechanisms played by Nrf2/Keap-1 pathway in Ectoine mediated effects in keratinocytes. Figure 4A shows that Ectoine dose-dependently and significantly increased the Nrf2/Keap-1 ratio with a maximum effect was observed at 1.5 µM Ectoine concentration. It was also observed that 1.5 µM Ectoine favored the nuclear translocation of Nrf2 protein with the maximum expression of Nrf2 from the nuclear protein fraction observed at the 2 h time point (Figure 4B). Data obtained from the immunofluorescence staining of HaCaT cells has also supported this effect (Figure 4D).

In human melanocytes and keratinocytes, Marrot et al. and others have explained the importance of Nrf2 defensive pathway in photo-oxidative stress responses [32]. We too studied the effect of Ectoine mediated antioxidant protein expression in HaCaT cells. Our time curve data indicated that Ectoine mediated expression of all three anti-oxidant proteins (HO-1, NQO-1, γ-GCLC), and Nrf2 were shown to express in a biphasic manner with the increasing time (0.5–12 h) with an observable effect was noted at 4 h time point (Figure 5A). From this, a concentration curve that measures the effect of Ectoine concentration on antioxidant protein expression was also determined at 4 h time point. Figure 5B shows that in comparison to the untreated cells, Ectoine treatment has dose-dependently increased the expression of HO-1, NQO-1, γ-GCLC proteins. We also measured how Ectoine concentration exhibited protective effects in HaCaT cells that were exposed to UVA radiation. Western blot data showed that Ectoine dose-dependently increased the expression of anti-oxidant proteins with a dramatic upregulation in the Nrf2/Keap-1 ratio as well (Figure 5C,D). These results indicated that Ectoine pretreatment (1.5 µM, 4 h) has the potential effect to induce antioxidant protein expression in HaCaT cells that could counteract the deleterious effects posed by UVA exposure.

Later, we determined the signaling pathways that mediated the activation of nuclear translocation of Nrf2 as well as the expression of anti-oxidant proteins. Our pharmacological inhibitor data revealed that p38, PI3/AKT, PKC, and CKII pathways were involved in the Ectoine mediated activation of nuclear NRf2. This data was consistent with the antioxidant expression data as well which showed that pharmacological inhibition of these four pathways down-regulated the expression of anti-oxidant proteins (HO-1, NQO-1, γ-GCLC). Except for the AKT activation (p-AKT), all three pathways (p38, PKC, and CKII) were activated at longer time points (after 30 min). The AKT pathway was demonstrated to be the first pathway activated (within 15 min) after exposure to Ectoine (Figure 6A–C).

Nrf2 knock-down technique has helped us to further demonstrate and confirm the key role played by Nrf2 in Ectoine mediated antioxidant and anti-melanogenic effects in HaCaT cells. Data showed that compared to the control siRNA cells, Nrf2 knockdown cells exposed to 1.5 µM Ectoine exhibited significant downregulation in the expression of HO-1, NQO-1, γ-GCLC antioxidant proteins (Figure 7A). On the other hand, these knockdown cells pre-treated with Ectoine and exposed to the UVA radiation did not affect the α-MSH expression, which confirmed that Nrf2 plays a key role in α-MSH expression in HaCaT cells (Figure 7B). In addition to the α-MSH expression, our DCF fluorescence data also revealed that Nrf2 knockdown is involved in the regulation of intracellular ROS production in UVA irradiated HaCaT cells that were pre-treated with 1.5 µM Ectoine (Figure 7C,D).

## 5. Conclusions

From the above data, we concluded that low concentrations of Ectoine (0.5–1.5 µM) could downregulate α-MSH and melanin production via the suppression of POMC and tyrosinase pathway in UVA irradiated HaCaT cells, indicating its anti-melanogenesis efficacy. Additionally, Ectoine was also involved in the suppression of intracellular ROS production in HaCaT cells. Unlike HaCaT cells, high concentrations of Ectoine (50–400 μM) were able to show the similar effect in B16F10 melanoma cells that have signified the fact that keratinocytes could play a key role in the Ectoine mediated anti-melanogenesis and skin-whitening effects in skin cells. Most importantly, Ectoine mediated beneficial effects via the activation of the Nrf2 pathway, that induces the expression of antioxidant proteins HO-1, NQO-1, and γ-GCLC. AKT was shown to be the first signaling pathway that initiates the activation of Nrf2 followed by the other pathways (p38, PKC, and CKII). Finally, silencing of Nrf2 directly provided the evidence that Nrf2 plays a key role in the regulation of intracellular ROS as well as the α-MSH production. We concluded that the main whitening mechanism of Ectoine should be reasoned by the inhibition of ROS-p53/POMC-α-MSH pathway in UVA-irradiated HaCaT cells. Therefore, Ectoine or its derivatives could be an active ingredient in the moisturizers and lotions that are used as potential and natural-based skin whitening agents in the cosmetic industry.
